# Tuning plasmid DNA amounts for cost-effective transfections of mammalian cells: when less is more

**DOI:** 10.1007/s00253-024-13003-x

**Published:** 2024-01-11

**Authors:** Aida Carreño, Rubén Guerrero-Yagüe, Enriqueta Casal, Rosa Mendoza, José Luis Corchero

**Affiliations:** 1 Institut de Biotecnologia i de Biomedicina, Universitat Autònoma de Barcelona, 08193 Bellaterra, Barcelona, Spain; 2 Present Address: Institut de Ciència de Materials de Barcelona, ICMAB-CSIC, Campus UAB, 08193 Bellaterra, Spain; 3 Present Address: Gene Therapy for Neurometabolic Disorders, Edifici H, Institute of Neurosciences (INc) & Department of Biochemistry and Molecular Biology, Universitat Autònoma de Barcelona, 08193 Bellaterra, Barcelona, Spain; 4 Present Address: Alderley Analytical Ltd. Alderley Park, Macclesfield, Cheshire, SK10 4TG UK; 5 CIBER de Bioingeniería, Biomateriales y Nanomedicina, Instituto de Salud Carlos III, 08193 Bellaterra, Barcelona, Spain; 6 Departament de Genètica i de Microbiologia, Universitat Autònoma de Barcelona, 08193 Bellaterra, Barcelona, Spain

**Keywords:** Polyethylenimine, Transfection, Transient gene expression, HEK 293F cells, Recombinant protein

## Abstract

**Abstract:**

Transient gene expression (TGE) in mammalian cells is a well-known approach to the fast expression of recombinant proteins. The human cell line HEK (human embryonic kidney) 293F is widely used in this field, due to its adaptability to grow in suspension to high cell densities in serum-free media, amenability to transfection, and production of recombinant proteins in satisfactory quantities for functional and structural analysis. Amounts of plasmid DNA (pDNA) required in transfections for TGE remain high (usually 1 µg pDNA/mL, or even higher), representing a noticeable proportion of the overall cost. Thus, there is an economic need to reduce amounts of coding pDNA in TGE processes. In this work, amounts of both pDNA and transfecting agent used for TGE in HEK 293F cells have been explored in order to reduce them without compromising (or even improving) the productivity of the process in terms of protein yield. In our hands, minimal polyethyleneimine (PEI) cytotoxicity and optimum protein yields were obtained when transfecting at 0.5 µg pDNA/mL (equal to 0.5 µg pDNA/million cells) and a DNA-to-PEI ratio of 1:3, a trend confirmed for several unrelated recombinant proteins. Thus, carefully tuning pDNA and transfecting agent amounts not only reduces the economic costs but also results in higher recombinant protein yields. These results surely have a direct application and interest for the biopharmaceutical industry, always concerned in increasing productivity while decreasing economic costs.

**Key points:**

*• Mammalian cells are widely used to produce recombinant proteins in short times.*

*• Tuning DNA and transfecting agent are of great interest to optimize economic costs.*

*• Reducing DNA and transfecting agent amounts result in higher protein yields.*

**Graphical Abstract:**

**Figure:**
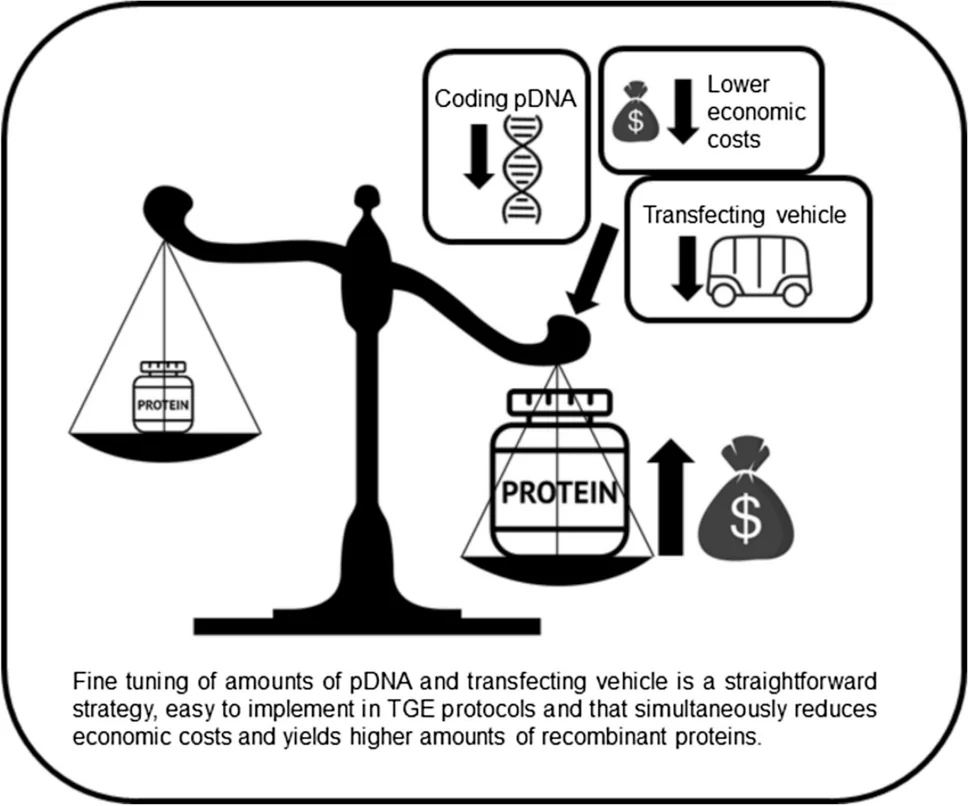

## Introduction

The introduction into cultured mammalian cells of a foreign gene and its further transient expression for a short period of time is a standard procedure routinely applied to the production of recombinant proteins. Compared to the tedious, time-consuming construction of stable clones constitutively expressing the protein of interest, transient gene expression (TGE) is preferred when many proteins (or several variants of a single protein) must be rapidly explored as putative biopharmaceuticals. In this context, TGE is a well-suited approach to fill this need, due to its ability to produce significant amounts of proteins within a short period of time (Wurm and Bernard 1999; Pham et al. 2006), and very convenient in early stages of the development of a new biopharmaceutical when several candidates need to be tested. TGE is based on the transfection of cells with a plasmid DNA (pDNA) carrying the transgene of interest, by means of different transfection reagents, and the subsequent generation of a heterogeneous population of transfected and non-transfected cells. Since the transgene is not integrated into the cell genome but it is expressed episomally, one main drawback of TGE is the loss of transgene expression over time, due to its dilution after each cell division (Middleton and Sugden 1992; Wade-Martins et al. 1999). Thus, after 2 to 6 days post-transfection (depending on the specific cell line and recombinant product), the synthesis of the protein of interest is stopped. On the other hand, one clear TGE advantage is the speed and versatility with which recombinant proteins can be produced. Due to these properties, TGE is not only one of the most used technologies for the production of recombinant proteins, especially in early development phases, but it is also gaining importance in the development of large-scale processes for novel products such as viral vectors for gene therapy (Merten et al. 2014) or different proteins (Baldi et al. 2007; O’Flaherty et al. 2020). Polyethyleneimine (PEI) is a stable cationic polymer with ethylenimine motifs responsible for its net positive charge. Characterized by a low cost and a high transfection efficiency, PEI is an ideal transfection reagent for TGE and large-scale productions. The ability of PEI to function as DNA carrier is based on the abundant amino nitrogen atoms in its structure, which enable it to bind to phosphate atoms in the negatively charged DNA. Thus, positively charged PEI and negatively charged DNA form stable complexes when mixed. Such complexes bind to the cell surface, where they are uptaken via endosomal vesicles, which release the plasmid to the cytoplasm. It has been described that recombinant protein yield is greatly affected by several variables such as transfection efficiency and cell culture conditions (e.g., the DNA/PEI ratio used and serum concentration in the culture medium and culture temperature). Therefore, in order to make TGE approaches more efficient for industrial purposes, great efforts have been addressed to improve aspects like efficient transfection of large volumes, simplification or reduction of handling steps, and optimization of pDNA amounts. A drawback of TGE approach is the amount of needed pDNA, typically 1 mg (or more) per liter of transfection, representing a significant proportion of the overall cost of TGE. Thus, there is an obvious economic need and interest to reduce pDNA without compromising the process productivity. Moreover, a pDNA reduction with its corresponding PEI reduction could have a collateral positive side-effect in terms of a lower cell cytotoxicity, since PEI is known to be toxic to cells when used at high concentrations (Florea et al. 2002; Breunig et al. 2007; Kafil and Omidi 2011). Therefore, concentrations and pDNA-to-PEI ratios need to be carefully optimized to obtain an adequate balance between PEI toxicity, cell viability, protein expression levels, and overall economic cost. In this context, it has been reported that some of the coding pDNA can be replaced by non-specific DNA (“filler DNA”) in PEI-mediated transfections of mammalian cells without a significant reduction in recombinant protein production (Kichler et al. 2005; Rajendra et al. 2012). With this in mind, and with the aim to further optimize TGE processes, we have explored in this work the possibility of tuning the amounts of codifying pDNA (focusing on its reduction without the need of replacing it with a filler DNA), and accordingly, the amounts of PEI and the impact of such change in the overall productivity of the process.

## Material and methods

### Design, synthesis, and cloning of recombinant genes

The vector pEGFP-C1 (Clontech, Palo Alto, CA) expressing the “enhanced green fluorescent protein” (GFP) was used to set up initial transfection conditions (Corchero et al. 2011) and for some experiments of this work. Sequences of the human major vault protein (MVP, UniProt Q14764, MVP_HUMAN) and of human beta-glucocerebrosidase (GCase, UniProt P04062, GLCM_HUMAN) were used to design the recombinant MVP-H6 and GCase-H6 genes, respectively. At the 3′ end of their native sequences, extra triplets encoding a six-histidine tag (H6) were added for further purification and detection purposes. These sequences were synthesized and cloned (by GeneArt®, Life Technologies) into the pTriEx1.1-Hygro vector (Novagen, cat. no. 70928–3, 6951 bp), between *Nco*I and *Eco*RI restriction sites. The resulting plasmids pTriEx1.1-MVP-H6 and pTriEx1.1-GCase encode the proteins MVP-H6 (899 amino acids and molecular weight of 100.1 kDa) and GCase-H6 (542 amino acids and molecular weight of 60.5 kDa), respectively. To express the human enzyme alpha-galactosidase A (GLA, UniProt P06280, AGAL_HUMAN), the vector pOpinE-GLA was used. This expression vector contains a gene encoding a full-length version of the GLA enzyme, cloned by the in-fusion methodology into vector pOpinE (Berrow et al. 2007), a derivative of vector pTriEx2 (Novagen). In this expression vector, GLA production is under the control of CMV immediate-early enhancer fused to the chicken β-actin (CBA) promoter and contains a His-tag fused to its C-terminus. Another expression vector also carrying wild-type GLA gene (pCIneo-GLA) was constructed by DNA synthesis (by GenScript, https://www.genscript.com/) of an *Xho*I-*Xba*I restriction segment containing the full-length coding sequence of GLA followed by a His-tag coding sequence and further cloning into pCI-neo vector (Promega Corp.; Madison, WI, USA) between *Xho*I and *Xba*I restriction sites. Using this pCIneo-GLA vector as a starting point, several vectors carrying mutated versions of GLA gene were generated (GenScript) by deletion or point mutations. In all these pCIneo-based expression vectors, protein expression is under the control of the human cytomegalovirus (CMV) immediate-early enhancer/promoter region and contains a His-tag at the C-terminus for detection purposes. The *E. coli* DH5α strain was used for the maintenance and amplification of these expression plasmids. Table 1 summarizes plasmids and proteins used in this work, including the GenBank accession number for their nucleotide encoding sequences.

**Table 1:** List of plasmids and recombinant proteins used in this work. All these proteins, except eGFP, contain a 6xHis-tag at their C-terminus for detection and purification purposes. For GLA protein and its mutants, size of unglycosilated monomer is shown. The final, active form for GLA is a highly glycosylated homodimer of approx. 105 kDa

Plasmid	Protein	Protein characteristics (size)	GenBank accession number
pEGFP-C1	eGFP	Enhanced green fluorescent protein (27 kDa)	AAB02576.1
pTriEx1.1-MVP-H6	MVP-H6	“Major vault protein,” main structural component of eukaryotic vault (100 kDa)	OR371540
pTriEx1.1-GCase	GCase-H6	Human beta-glucocerebrosidase, responsible for Gaucher disease (60 kDa)	OR371541
pOpinE-GLA	GLA-His	Human alpha-galactosidase A, responsible for Fabry disease (50 kDa)	OR371542
pCIneo-GLA	GLA-His	Human alpha-galactosidase A (50 kDa)	OR371531
pCIneo-GLA-C90T	GLA C90T	Human alpha-galactosidase A with mutation C90T (50 kDa)	OR371532
pCIneo-GLA-N139S	GLA N139S	Human alpha-galactosidase A with mutation N139S (50 kDa)	OR371533
pCIneo-GLA-R252T	GLA R252T	Human alpha-galactosidase A with mutation R252T (50 kDa)	OR371534
pCIneo-GLA-CN	GLA CN	Human alpha-galactosidase A with mutations C90T and N139S (50 kDa)	OR371535
pCIneo-GLA-CR	GLA CR	Human alpha-galactosidase A with mutations C90T and R252T (50 kDa)	OR371536
pCIneo-GLA-NR	GLA NR	Human alpha-galactosidase A with mutations N139S and R252T (50 kDa)	OR371537
pCIneo-GLA-D7	GLA D7	Human alpha-galactosidase A with deletion of 7 residues (at C-terminus) (49 kDa)	OR371538
pCIneo-GLA-D10	GLA D10	Human alpha-galactosidase A with deletion of 10 residues (at C-terminus) (48.5 kDa)	OR371539

### Cell culture and transfection

Recombinant proteins were expressed by polyethylenimine (PEI)-mediated transfection and further transient gene expression (TGE) in the suspension-adapted human embryonic kidney (HEK) cell line FreeStyle 293-F (Invitrogen, Life Technologies, ref. R790-07). These cells were maintained and grown according to the manufacturer’s instructions in Erlenmeyer flasks with plain bottoms and vented caps in the FreeStyle™ expression medium (Thermo Fisher Scientific, ref. 12,338,018), at 37 °C in an 8% CO_2_ atmosphere with gentle shaking (120 rpm). One day before transfection, cells were subcultured to a density of 0.5 × 10^6^ cells/mL. On the day of transfection, cells were checked to be at ~ 1 × 10^6^ cells/mL and were dispended in 6-well plates (2 mL cells/well). Cells were transfected using 25 kDa linear, transfection-grade PEI (Polysciences, ref. 23,966–100). Briefly, pDNA and PEI were mixed in sterile pre-warmed medium and incubated 10 min at room temperature. Initial transfection conditions for this cell line had been previously set and optimized (Corchero et al. 2011) at 1 µg pDNA/mL of culture (equivalent to 1 µg pDNA/million cells) and a ratio DNA to PEI of 1:3 (w/w). To optimize transfection conditions, amounts of pDNA were varied as stated for each experiment/condition, but always maintaining the same ratio DNA to PEI of 1:3. The final volume of polyplexes mixture was 10% of the total volume of culture to be transfected. After incubation, pDNA-PEI polyplexes were added dropwise to cells. In all cases, valproic acid (VPA, Merck Sigma-Aldrich ref. P4543) was added to cells (4 mM final concentration) 4 h post-transfection to improve recombinant protein expression.

### Cell viability

Cell viability was estimated by using the Trypan blue exclusion assay. This assay is based on the principle that live, and viable cells possess intact cell membranes that can exclude certain dyes, such as trypan blue. On the contrary, dead cells do not. In this assay, 10 µL of cell suspension is mixed with 10 µL of dye and then visually examined at the microscope (Nikon Eclipse Ts2) to determine whether cells take up (blue cells, dead) or exclude dye (white cells, viable).

### SDS-PAGE and western blot analyses

To check protein expression, samples from cell cultures were taken 5 days after transfection (unless otherwise stated), centrifuged (13,400 rpm, 10 min), and after that, supernatants and cell pellets were separated by further analysis by SDS-PAGE. Supernatants were used to follow the expression of secreted proteins, while total cell pellets were resuspended in phosphate-buffered saline (PBS) and further used to explore expression levels for intracellular proteins. To detect recombinant proteins, SDS-PAGE was performed by using TGX Stain-Free™ FastCast™ acrylamide 12% (Bio-Rad, ref. 161–0185) and further visualization of proteins with a ChemiDoc™ Touch Imaging System (Bio-Rad). Western blot analyses were also performed. To visualize immunoreactive bands, an anti-His mouse monoclonal antibody (Clontech, ref. 631,212) was used as primary Ab to detect MVP-H6 and GCase-H6 proteins, an anti-GFP mouse monoclonal antibody (Santa Cruz Biotechnology, ref. sc-9996) to detect eGFP protein, and an anti-GLA rabbit polyclonal antibody (Sigma, ref. HPA000237) to detect GLA enzyme and its mutants. Samples to be quantitatively compared were run in the same gel and processed as a set. Densitometric analyses of the immunoreactive bands were performed with the Image Lab™ software (version 5.2.1., Bio-Rad).

### Microscopy images

Microscopy images were taken in a Nikon Eclipse Ts2 directly on 6-well plates containing transfected HEK 293F cells, without any processing or preparation.

## Results

### Effect of pDNA amounts on expression of human GLA enzyme and its mutants

**Fig. 1:**
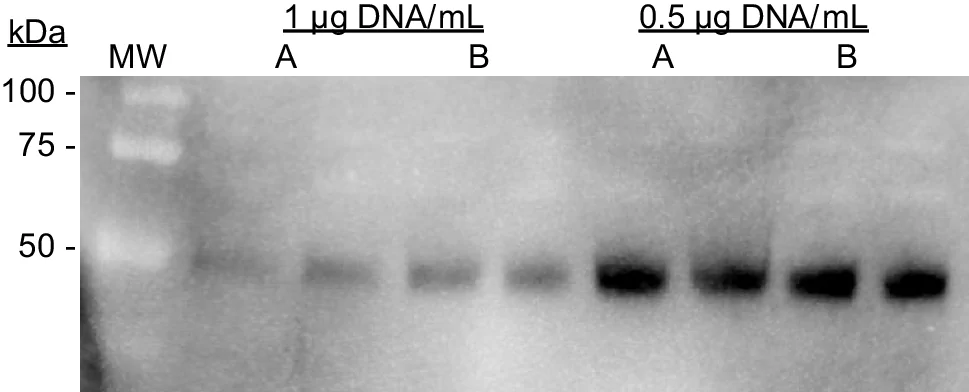
Comparison of GLA expression by HEK 293F cells transfected at 1 or 0.5 µg pDNA/mL. Two independent cultures (A and B) for each condition were tested, and each culture was loaded in the gel by duplicate. Legend: MW, molecular weight marker

In an attempt to optimize pDNA amounts used in our TGE protocols for GLA enzyme production, we compared the original conditions (1 µg pDNA/mL) with 0.5 µg pDNA/mL, maintaining the ratio DNA to PEI of 1:3. The novelty of this approach compared to previous ones describing similar experiments (Kichler et al. 2005; Rajendra et al. 2012) is that, in our case, no filler DNA was added in substitution of the eliminated GLA-encoding plasmid. The goal of the experiment was to determine if a percentage of the coding pDNA could be eliminated (with no replacement) without a significant reduction in recombinant protein production. Results (Fig. 1) showed that not only the reduction of a percentage of pDNA was possible and not detrimental for protein yield, but that amounts of secreted GLA enzyme were clearly higher (a 2.5 + / − 0.19 fold increase, according to the densitometric analysis of the western blot image) when transfecting with half (0.5 µg pDNA/mL) of the standard DNA amount (1 µg pDNA/mL).

**Fig. 2:**
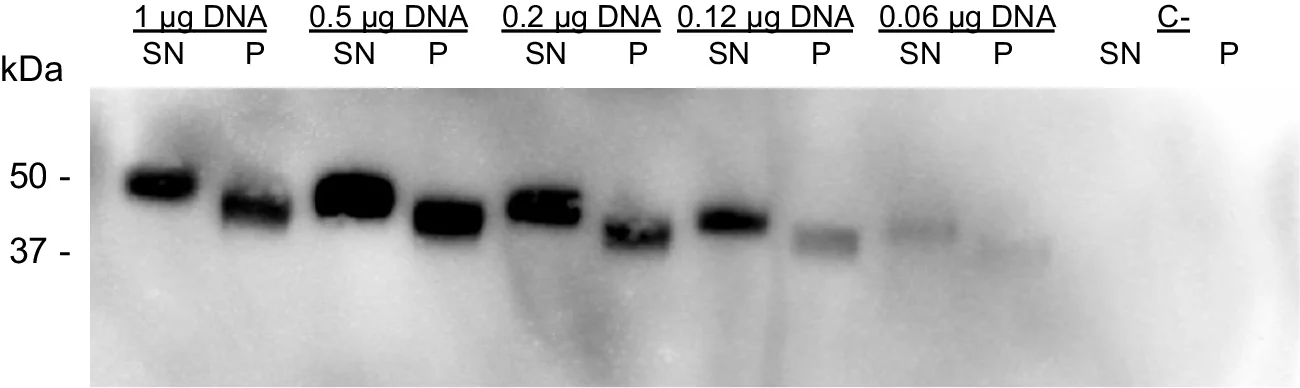
GLA enzyme amounts in supernatants (SN) and cell pellets (P) from HEK 293F cell cultures transfected with the indicated concentrations of pDNA/mL. Legend: C- indicates a negative control (non-transfected cells)

Even though the promising results were obtained, it was not clear if GLA enzyme was better expressed, or whether it was equally expressed but better secreted when transfecting at 0.5 µg pDNA/mL. Moreover, these results prompted us to test if it was possible to reduce even more the pDNA amounts used and still obtain equal (or better) protein amounts. For that, in a second experiment, we tested a wider range of pDNA (from 1 to 0.062 µg pDNA/mL) with the aim of testing not only the amount of secreted protein in each condition but also the amount of protein retained within the intracellular fraction. Five days post-transfection, cells and supernatants were separated and analyzed by SDS-PAGE and western blot. Results (Fig. 2) confirmed that transfecting cells with 0.5 µg pDNA/mL increase (a 2.4-fold increase, according to the densitometric analysis of the western blot image) amounts of both secreted and intracellular GLA enzyme. However, decreasing even more the amount of pDNA did not result in a further increase in GLA expression and accumulation (neither in the supernatant nor in the cell pellet). GLA solubility and secretion levels were not affected by transfection conditions, since percentage of enzyme secreted to the medium was maintained in all cases at values near 60% (59 + / − 4.1), indicating that GLA enzyme is better synthesized, but not better secreted.

**Fig. 3:**
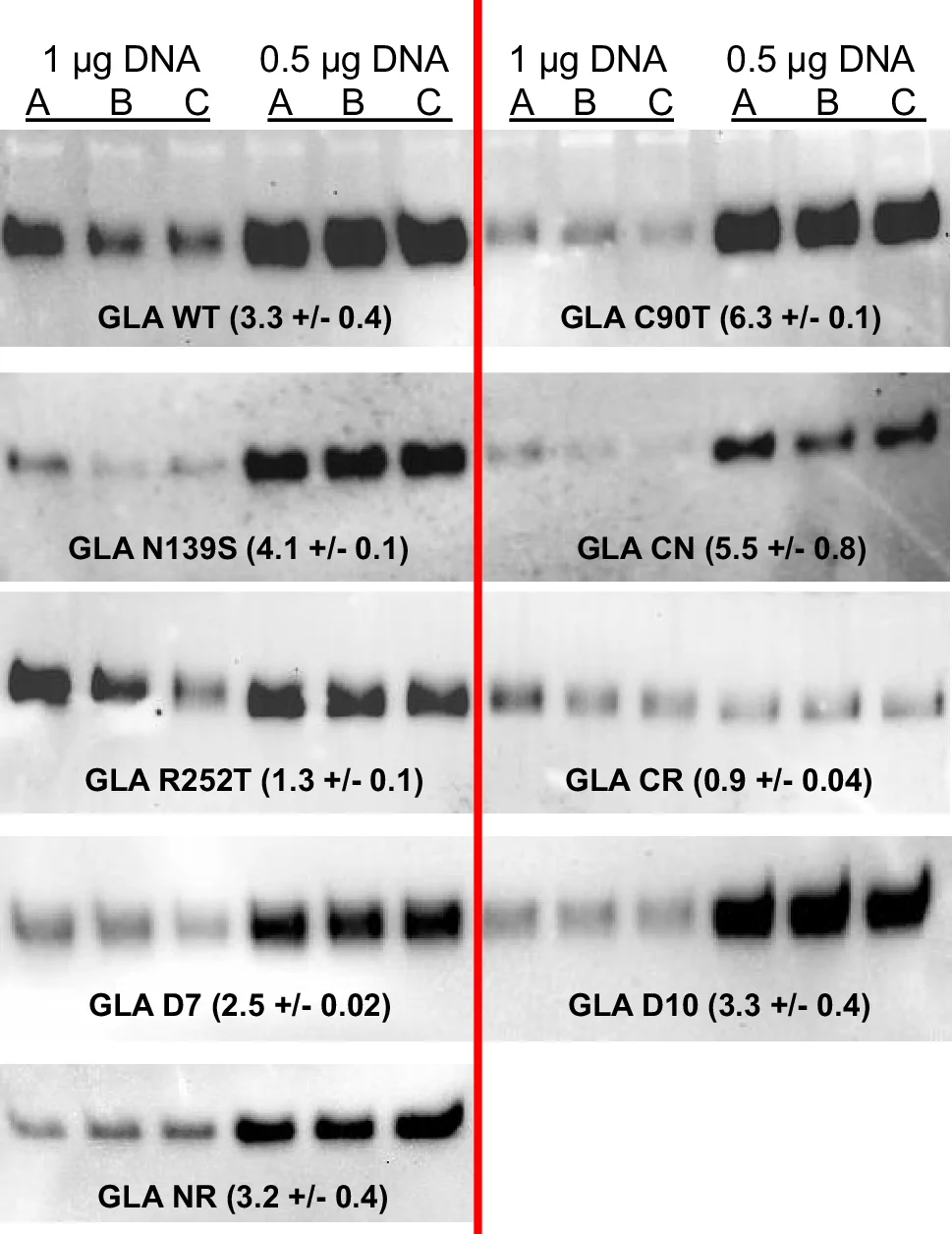
Comparison of GLA expression by HEK 293F cells transfected at 1 or 0.5 µg pDNA/mL. Samples from 3 independent cultures (A, B, and C) for each protein and condition were analyzed. For each protein, the fold increase in its protein yield is indicated (mean and standard error of the three replicas)

These results had been obtained by transiently expressing GLA enzyme under the control of the CMV immediate-early enhancer fused to the chicken β-actin (CBA) promoter, the regulatory elements present in the pOpinE expression plasmid. To rule out a putative effect of the specific promoter strength, wild-type GLA enzyme and several GLA mutants were expressed under the control of a different promoter, the human cytomegalovirus (CMV) immediate-early enhancer/promoter present in pCI-neo plasmid. In this case, and considering previous results, only two transfection conditions (1 and 0.5 µg pDNA/mL) were tested. Results (Fig. 3) showed that, again, amounts of secreted enzyme were clearly higher when transfecting at 0.5 µg pDNA/mL. The improvement was observed not only for wild-type enzyme but also for its mutants (ranging from 1.3-fold increase for mutant R252T to a 6.3-fold increase for C90T mutant). The only exception was the double mutant GLA-CR, which in general was poorly expressed and showed no increase in protein yield under the new transfection condition.

### Effect of pDNA amount on expression of other recombinant proteins

**Fig. 4:**
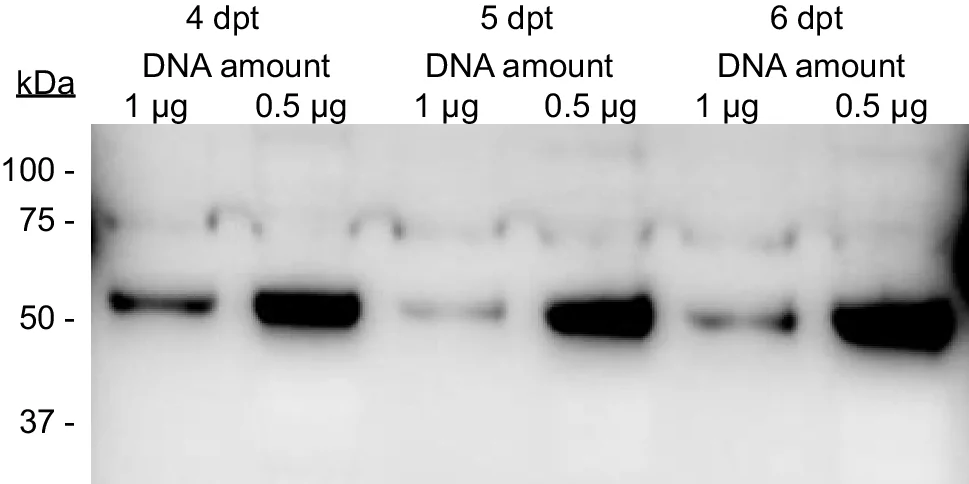
Comparison of GCase expression by HEK 293F cells transfected at 1 or 0.5 µg pDNA/mL. Samples (total cell pellets) taken on days 4, 5, and 6 post-transfection were analyzed

To explore if this effect is exclusive of the GLA enzyme or could be translated to the expression of other recombinant proteins, we explored the effect of reducing amounts of pDNA in the expression of another lysosomal enzyme, the human beta-glucocerebrosidase (GCase). In this case, previous experiments had indicated that when overexpressed in HEK 293F cells, GCase enzyme is mainly found in its insoluble, intracellular form (data not shown). Results (Fig. 4) confirmed that, in the case of GCase enzyme, lower amounts of pDNA yielded higher amounts of enzyme, at all days post-transfection analyzed (for this protein, a 5.7 + / − 1.6 fold increase, according to the densitometric analysis of the western blot image).

**Fig. 5:**
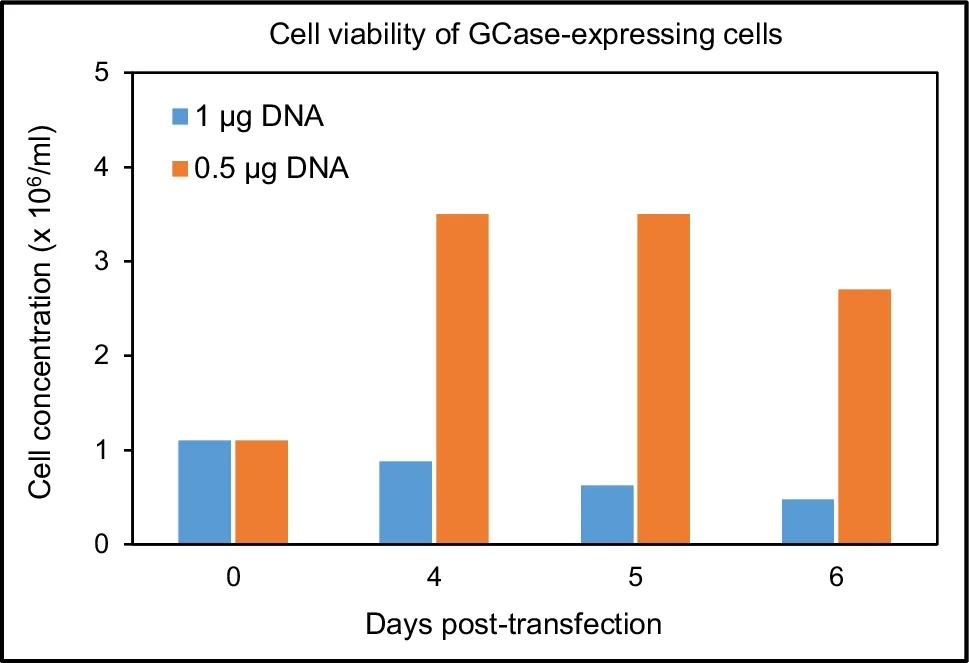
Cell viability of HEK 293F cells expressing GCase enzyme at different transfection (1 or 0.5 µg pDNA/mL) conditions

Since PEI used as transfection agent is known to exert some toxicity over eukaryotic cells, we checked the cell viability after transfection. Results (Fig. 5) indicated that cells transfected at 0.5 µg pDNA/mL maintained good viability after transfection, growing up to approx. 3.5 × 10^6^ cells/mL. On the contrary, transfection at the original condition of 1 µg pDNA/mL resulted in a clear deleterious effect on cell viability, with a decrease of cell concentration down to 0.5 × 10^6^ cells/mL. Since the ratio DNA to PEI of 1:3 had been maintained constant in both cases, more pDNA also meant more PEI and, consequently, more cell toxicity. This has a clear effect on cell viability, which, in turn, results in less viable, producing cells during transient gene expression.

**Fig. 6:**
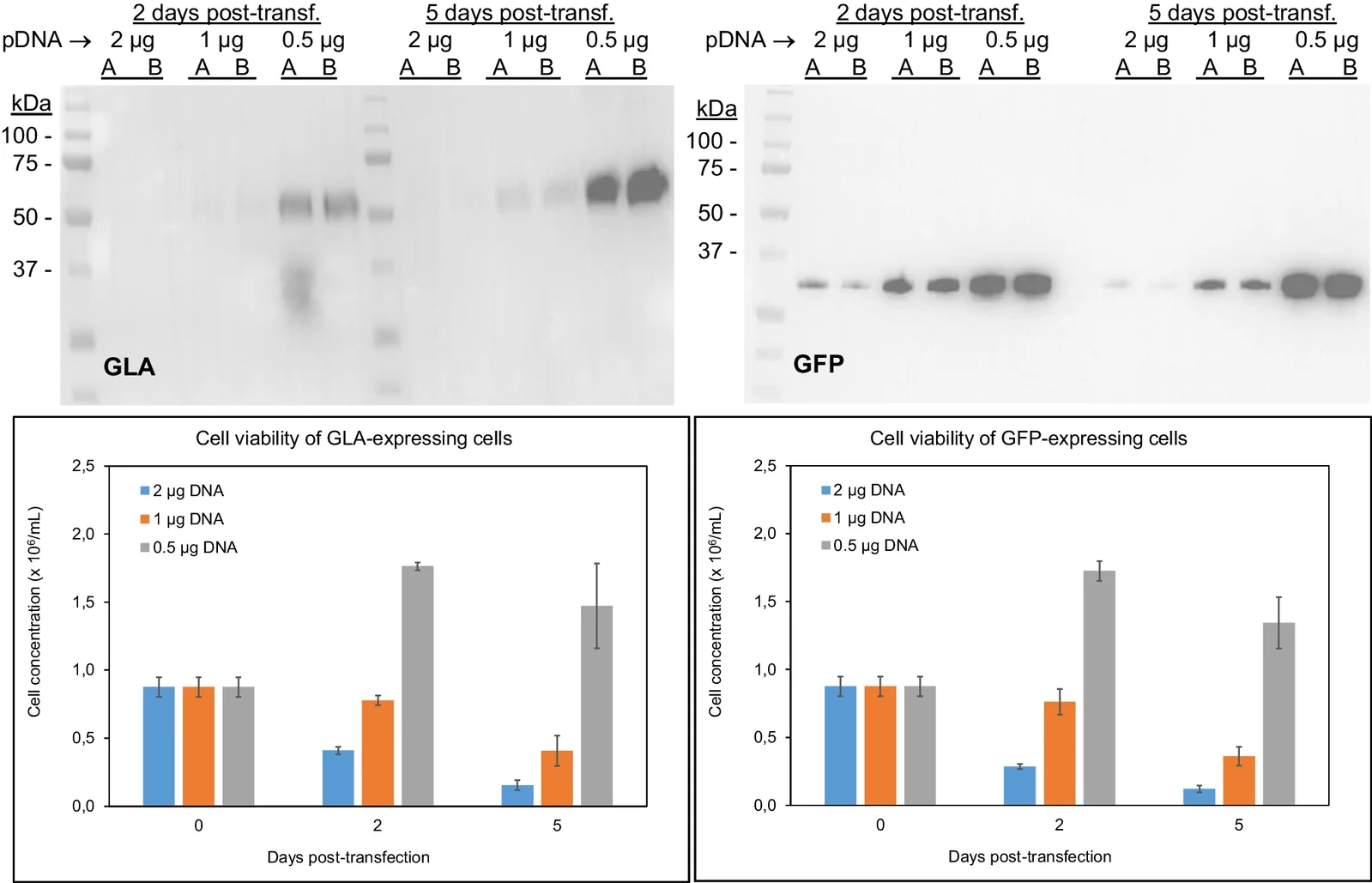
Amounts of expressed recombinant protein (top) and cell viability (bottom) of HEK 293F cells expressing GLA enzyme (left) and GFP (right) at different transfection conditions (2, 1, or 0.5 µg pDNA/mL). Two independent cultures (A and B) were used to express each recombinant protein. Cell viability results shown represent the mean and standard error of the mean (SEM) of two independent samples analyzed by duplicate

To confirm the effect of transfection conditions on cell viability, we expressed again lysosomal enzyme GLA and the fluorescent protein GFP, checking simultaneously in both cases the amounts of protein expressed and the cell viability at different times (2 and 5 days) after transfection. Results (Fig. 6) confirmed that amounts of pDNA clearly correlated with cell viability. In this line, transfecting at 0.5 µg pDNA/mL and the better cell viability associated with this condition resulted in a clearly higher protein expression. Such increase was observed already at 2 days post-transfection and ranged from a 2.7-fold increase for GFP to almost a tenfold increase for GLA enzyme.

**Fig. 7:**
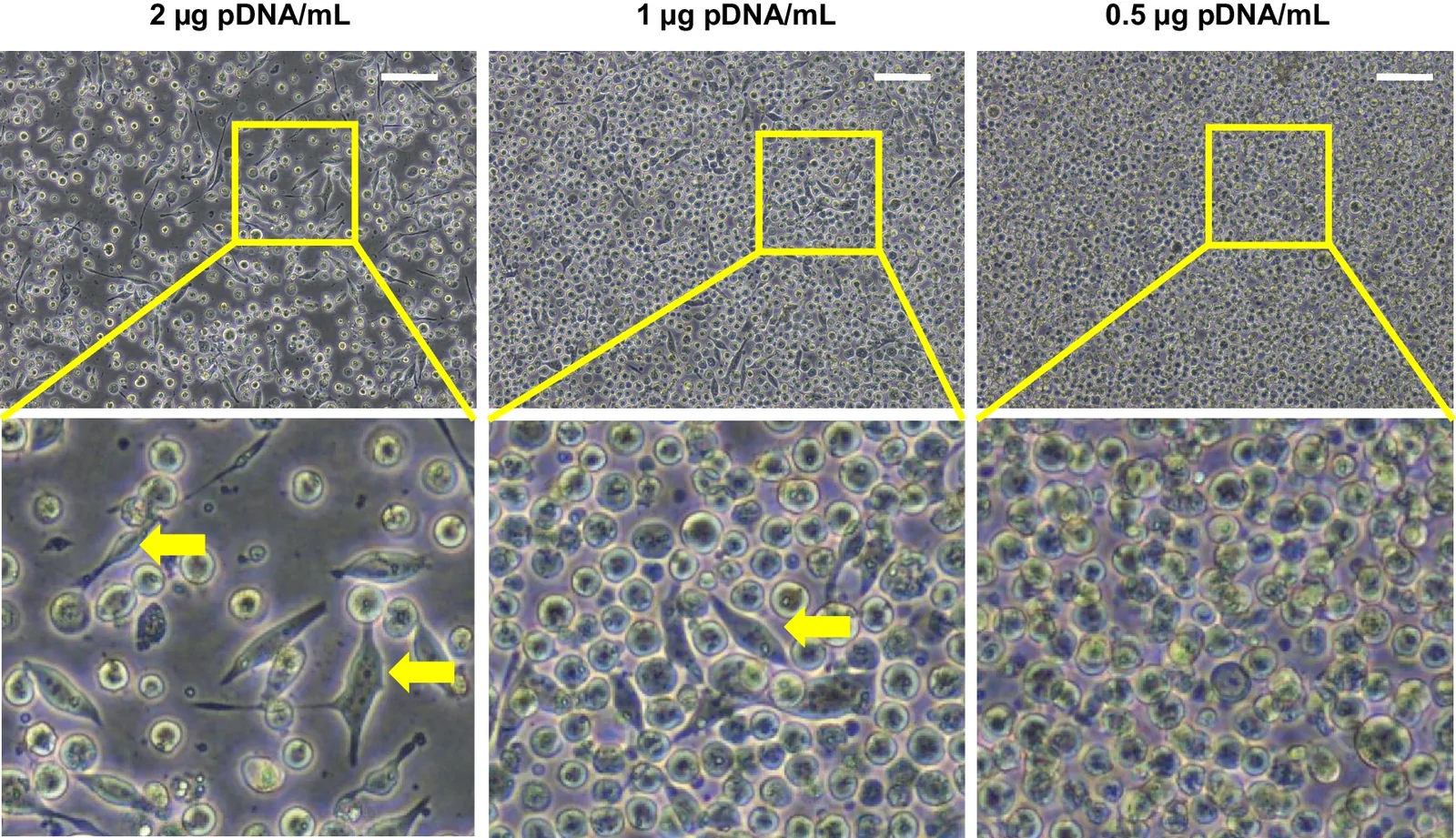
Microscopic images of cultures of HEK 293F cells expressing MVP-H6 protein taken 5 days post-transfection at 2, 1, or 0.5 µg pDNA/mL. Areas within yellow squares are shown in detail in bottom images. Yellow arrows point out some cells showing abnormal morphologies. Scale bars are shown in top images: 100 µm

As an example of another recombinant protein, we expressed a His-tagged version of the human “major vault protein” (MVP), the main component of the intracellular organelle known as “vault” (Anderson et al. 2007; Tanaka et al. 2009; Querol-Audí et al. 2009; Woodward et al. 2015). For that, HEK 293F cells were transfected with plasmid pTriEx1.1-MVP-H6, which codifies for MVP-H6 protein, according to previous work done in our laboratory (Martín et al. 2022). In this case, microscopy images of transfected cells revealed a clear effect on cell viability and morphology depending on the amount of pDNA/PEI added (Fig. 7). As previously seen, transfection with high amounts of DNA/PEI (especially at 2 µg pDNA/mL) resulted in lower cell viability, as confirmed by the lower cell concentration visible on the microscopy images. Also, cells with abnormal shapes were clearly detected, being more abundant when increasing the DNA/PEI concentration. On the contrary, when transfecting at 0.5 µg pDNA/mL, images revealed that cells maintained good viability, showing healthy, normal morphologies.

**Fig. 8:**
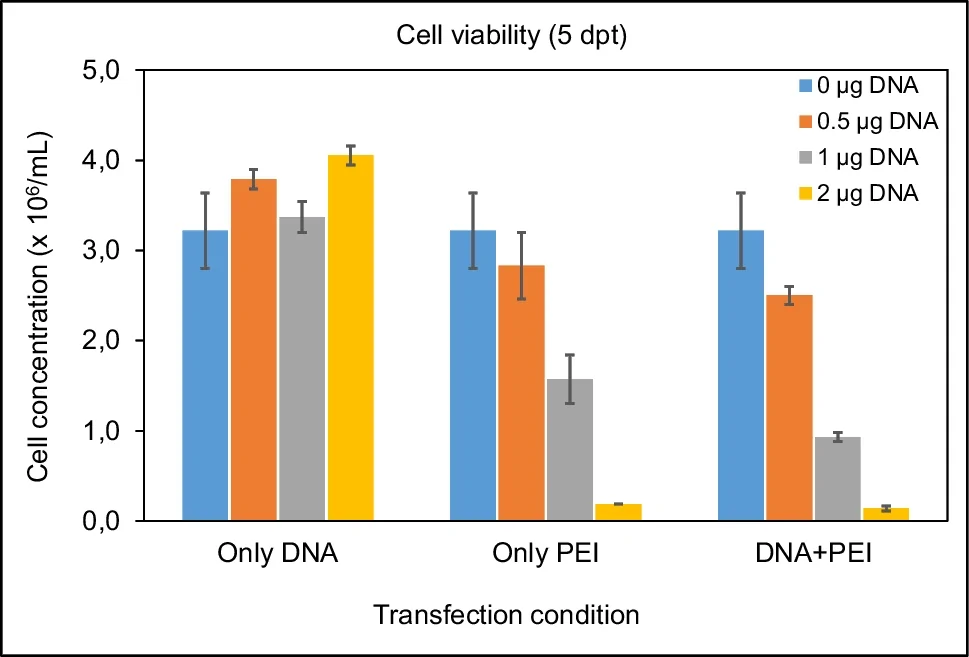
Cell viability of HEK 293F cells (at 5 days post-transfection) expressing the protein MVP-H6 under different transfection conditions. Results shown represent the mean and standard error of the mean (SEM) of two independent cultures for each condition

To confirm these results and to rule out a putative deleterious effect due to the pDNA added, experiment was repeated but included as a control mock transfections with only pDNA or only PEI. At 5 days post-transfection, cell viability was estimated for all conditions. Results (Fig. 8) showed that when cells were transfected with only pDNA, cell viability was comparable to that of control, untransfected cells, confirming the non-toxicity of this material. Also, we confirmed that higher amounts of PEI confer higher toxicity resulting in a clear decrease on cell viability, irrespective of the presence or not of DNA. All these results together confirmed that toxicity, low cell viability, and low expression levels were due to the amounts of PEI added and not by the amount of pDNA itself.

**Fig. 9:**
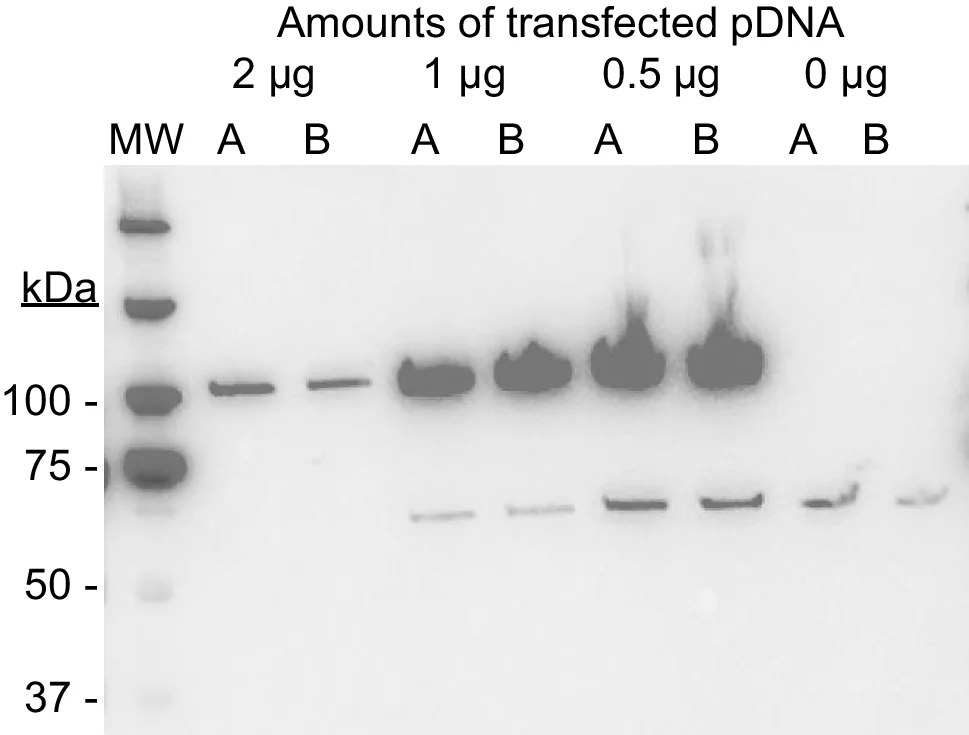
Amounts of expressed recombinant protein MVP-H6 by transfected HEK 293F cells at different transfection conditions (2, 1, or 0.5 µg pDNA/mL). Samples A and B correspond to two independent cultures for each condition

Cell lysates of transfected cells were also analyzed by SDS-PAGE and western blot, to check differences in the amounts of MVP-H6 protein expressed under all the tested transfection conditions. Again, clearly higher amounts of protein were obtained when decreasing the amounts of DNA/PEI used to transfect cells (Fig. 9), corroborating the previous results obtained for other proteins. In this case, a 1.4 + / − 0.03 fold increase (according to the densitometric analysis of the western blot image) was achieved when reducing the amount of transfected pDNA from 1 to 0.5 µg/mL.

## Discussion

Production of biopharmaceuticals in mammalian “cell factories” has been consistently gaining importance in the industry over the last years (Walsh 2018; Walsh and Walsh 2022). In this scenario, and according to the latest survey of biopharmaceutical approvals (corresponding to the period from January 2018 to June 2022), mammalian cells continue to be the most often used expression system. Of the 159 approved recombinant products expressed in cell-based systems, 67% are produced in mammalian cells. As in previous surveys, Chinese hamster ovary (CHO) cells continue to be the preferred choice, being used to produce 89% of those products expressed in mammalian systems (Walsh and Walsh 2022). This is mainly due to its ability to express recombinant proteins with post-translational modifications similar to that of human proteins (Harcum and Lee 2016). CHO cells constitutively expressing a recombinant protein for extended cultivation times (“stable clone”) are established by the integration of a foreign DNA within the host genome followed by a costly and time-consuming (up to several months) process to select, identify, and characterize the best-producer clones (Wurm 2004). Such efforts may be lost if the recombinant product finally does not fulfill the required specifications for its therapeutic use.

In contrast to this stable gene expression, transient gene expression (TGE) involves short-term (typically for up to 10 days post-transfection) protein production in the absence of genetic selection of pDNA. Thus, TGE in cultured mammalian cells is intensively used for the fast obtention of recombinant proteins in amounts enough to perform biochemical, biophysical, and pre-clinical studies. In this scenario, serum-free suspension-cultivated HEK 293F cells have become a widely used cell host for large-scale transient gene expression.

Standard TGE procedure requires first the complexation of the pDNA molecule with polycations such as polyethylenimines (PEIs), leading to the formation of “polyplexes,” followed by the incubation with target cells. PEI is a stable cationic polymer with ethylenimine motifs responsible for its net positive charge, moderately cytotoxic, and non-biodegradable and belongs to the most efficient non-viral gene transfer agents developed so far (Boussif et al. 1995). It has been successfully used to transfect a wide plethora of cell lines, both in vitro and in vivo (Boussif et al. 1995; Boeckle et al. 2004). The ability of PEI to function as DNA carrier is based on the abundant amino nitrogen atoms in its polymeric structure, which enable it to bind to phosphate atoms in the negatively charged DNA. Thus, the positively charged PEI and the negatively charged DNA forms stable DNA/PEI complexes when mixed that bind to the cell surface. Uptake of these complexes occurs via endosomal vesicles, which release the plasmid to the cytoplasm after osmotic swelling (Wightman et al. 2001). The endosomolytic activity of PEIs seems to be based on the ability to capture protons that enter the vesicle during acidification. This process results in a swelling of the endosomes that possibly facilitates membrane disruption. Thus, with low molecular weight, linear PEI is a cost-effective transfection reagent of particular interest for transfection of mammalian cells (Reed et al. 2006). Although many factors contribute to a successful transfection, both the PEI-to-DNA ratio used for polyplexes formation and the presence of non-complexed, free PEI plays critical roles in pDNA uptake and intracellular trafficking (Kircheis et al. 2001; Bieber et al. 2002; Boeckle et al. 2004; Honoré et al. 2005; Ketola et al. 2011). Importantly, effects of residual PEI on the downstream processing of proteins and on protein quality have not been systematically addressed. Other factors, including presence of serum or cell density, influence the efficiency of PEI-based transfection and consequently further TGE (Kuroda et al. 2009).

A second key factor in TGE processes is the required amounts of pDNA. Typical transfection protocols use 1–1.25 µg pDNA per mL culture (or even higher amounts), constituting a significant amount of pDNA and a major cost factor, especially for industrial large-scale TGE. Another issue related to the use of high amounts of pDNA in TGE processes is the elimination of contaminants such as bacterial RNA and endotoxin during pDNA purification (Stadler et al. 2004). Although it has been reported that these contaminants do not significantly affect the efficiency of transfection with calcium phosphate (Wright et al. 2003), the removal of lipopolysaccharide (LPS) is critical in downstream processing of the protein (Wakelin et al. 2006). Therefore, reducing pDNA amounts in TGE, without compromising the process productivity, is of great interest for the industry, and significant efforts have been addressed in this direction. First approaches in this scenario dealt with the possibility of replacing some coding pDNA with an irrelevant, “filler” DNA (usually, sheared herring sperm DNA). The rationale behind this was that transgene copy numbers achieved within PEI-transfected cells for TGE are much greater than those typically observed in stable CHO-derived cell lines (1 to several 100 transgene copies per cell), but surprisingly, such CHO stable cell lines show better volumetric productivities than those achieved by TGE (Wurm 2004). This observation suggested that not all the transgene copies successfully transfected into target cells are efficiently used for the cell machinery, thus opening the possibility of tuning (reducing) amounts of coding pDNA in TGE processes (reducing both economic costs and the risk of immune stimulation triggered by bacterial DNA). In a first study, it was confirmed that a significant percentage of pDNA can be replaced by filler DNA in PEI-mediated transfection of cultured mammalian cells. More than half of the pDNA could be substituted by filler DNA without significantly decreasing transfection efficiency both in cultured cells and after systemic administration in mice. However, all conditions tested resulted in levels of recombinant protein expression lower than those attained by transfecting with 100% of coding pDNA (Kichler et al. 2005). In another study further exploring this strategy, it was described that reduction of coding pDNA (replaced with filler DNA) and protein yields did not decrease proportionally. For example, in transfections of HEK-293E cells, reduction of pDNA to 33% or 17% from the optimal amount and its replacement with filler DNA resulted in recombinant protein yields of 75% and 65%, respectively, compared to transfections using a 100% of coding pDNA (Rajendra et al. 2012). Other conclusions of this study shedding light to this phenomenon were that filler DNA allows pDNA to be distributed over a greater number of DNA-PEI complexes, resulting in a higher percentage of transfected cells, and that the presence of filler DNA within complexes may also allow pDNA to be more efficiently utilized by the cell’s transcription machinery, resulting in a higher level of transgene mRNA.

Even though the partial replacement of coding pDNA by cheaper filler DNA without greatly compromising protein yields is in itself a significant improvement and advantage in the overall TGE process, all the conditions explored and described in the previous studies rendered lower amounts of the protein of interest compared to original condition (100% of coding pDNA). In the present work, the goal was to explore a new cost-effective approach in terms of tuning pDNA amounts in TGE processes without the need of adding filler DNA. Results showed that it is possible to reduce amounts of pDNA (and PEI) used to express several recombinant proteins in HEK 293F cells. Surprisingly, this reduction of amounts of both pDNA and PEI resulted in a significant increase in protein yield, improvement that was confirmed for different unrelated proteins. It is worthy to note that the experimental set-up and conditions to transfect cells used here differ in some parameters when compared with the abovementioned studies. For example, cell lines and pDNA and PEI amounts and ratios can vary within laboratories and studies. However, the highest transfection efficiencies have been previously described with a DNA-to-PEI ratio of 1:3 (w/w), corresponding to an N/P ratio (nitrogen residues/DNA phosphates) of 23, with HEK 293 s cells (de Los Milagros Bassani Molinas et al. 2014). Also, such DNA-PEI ratio of 1:3 has been reported to give the highest expression rates using suspension-adapted HEK-293EBNA cells (Baldi et al. 2005). This is coincident with our experimental set-up for transfections, where (irrespective of the amount of pDNA used) the ratio pDNA to PEI was always 1:3 (N/P ratio of 23).

On the other hand, medium changes before and/or after transfection, cell concentration before transfection, cell dilution after transfection, or applying mild hypothermia after transfection are common procedures applied to TGE processes, but none of them is needed in our procedure, which greatly simplifies the overall workflow. Our strategy avoids all these tedious, time-consuming steps, rendering an easy and straightforward protocol to transfect HEK 293F cells. The low amounts of pDNA and PEI used in this approach result in cells maintaining a good viability after transfection, comparable to that for non-transfected cells. As shown, PEI, when used at the highest concentrations, is responsible for the low concentration of viable, producing cells. Moreover, as reflected in microscopy images, a percentage of these “surviving” cells shows a clearly abnormal morphology, corroborating the deleterious PEI effect over the cells, and therefore, clearly affecting the overall productivity of the process. Taken together, the data stress the idea that transfections and TGE are very sensitive to PEI concentrations. Thus, PEI conditions should be well-defined for each cell line.

In addition to this, our results may also have other practical implications. When produced in bacteria, pDNA containing specific sequences can have immune stimulatory effects resulting in production of proinflammatory cytokines (IFN-γ and IL-12) (Yew et al. 2000; Loisel et al. 2001). This, in turn, was shown to be responsible for the inhibition of gene expression (Qin et al. 1997; Tan et al. 1999). Strategy proposed here based on the reduction of pDNA transfected could avoid, or at least mitigate, this immune stimulatory effect previously described.

As conclusion, the strategy presented here is a straightforward approach, easy to implement in any TGE protocol, and that simultaneously presents the clear advantages of reducing economic costs and yielding higher amounts of recombinant proteins. All together is of clear interest for laboratories or companies using this strategy to manufacture proteins of industrial or biomedical interest.

## Data Availability

All data generated and analyzed during this study are included and available in this article.
